# Predictors of Functional Outcome in Patients with Bipolar Disorder

**DOI:** 10.1192/j.eurpsy.2022.71

**Published:** 2022-09-01

**Authors:** A. Erfurth, G. Sachs

**Affiliations:** 1 Medical University of Vienna, Department Of Psychiatry And Psychotherapy, Vienna, Austria; 2 Klinik Hietzing, 1st Department Of Psychiatry And Psychotherapeutic Medicine, Vienna, Austria

**Keywords:** bipolar disorder, functional outcome, psychoeducation, Neurocognition

## Abstract

Introduction Bipolar disorder is a severe disorder that is often accompanied by deficits in both neurocognitive (1) and psychosocial function (2). At the Department of Psychiatry and Psychotherapy of the Medical University of Vienna we performed a study to further identify potential cognitive, clinical and treatment-dependent predictors for functional impairment, symptom severity and early recurrence in bipolar patients (3). Methods Forty-three remitted bipolar patients and 40 healthy probands were assessed with a cognitive battery. In a randomized controlled trial, remitted patients were assigned to two treatment conditions as add-on to state-of-the-art pharmacotherapy: cognitive psychoeducational group therapy (CPEGT) over 14 weeks or treatment-as-usual. At 1 year after therapy, functional impairment and severity of symptoms were assessed. Results As compared to healthy probands, bipolar patients showed lower performance in executive function, sustained attention, verbal learning and verbal fluency. Both attention and CPEGT predicted occupational functioning. In our study verbal memory recall was a predictor for symptom severity. Discussion Our data suggest that bipolar patients benefit from CPEGT in the domain of occupational life. Implications for treatment strategies are discussed. Solé B, Jiménez E, Torrent C, Reinares M, Bonnin CDM, Torres I, Varo C, Grande I, Valls E, Salagre E, Sanchez-Moreno J, Martinez-Aran A, Carvalho AF, Vieta E. Int J Neuropsychopharmacol (2017) 20:670–80. Tohen M, Zarate CA Jr, Hennen J, Khalsa HM, Strakowski SM, Gebre-Medhin P, Salvatore P, Baldessarini RJ. Am J Psychiatry (2003) 160:2099–107. Sachs G, Berg A, Jagsch R, Lenz G, Erfurth A. Front Psychiatry (2020) 23;11:530026.

**Disclosure:**

No significant relationships.

